# Early warning scores and peripheral perfusion index: A Comparative assessment for 30-day mortality in critical patients in emergency departments

**DOI:** 10.1007/s11845-026-04358-3

**Published:** 2026-04-10

**Authors:** Ahmet Celal Ozsoy, Huseyin Avni Demir, Mustafa Simsek, Ahmet Celik, Muhammed Tabası, Zahit Donmez, Abdul Samed Rayan, Eyyup Sabri Seyhanli

**Affiliations:** Dept of Emergency Service, University of Health Sciences, Mehmet Akif Inan Training and Research Hospital, Sanlıurfa, Turkey

**Keywords:** Critical illness, NEWS-2, MEWS, Peripheral perfusion index, Mortality prediction

## Abstract

**Background:**

Early identification of high-risk patients in emergency departments (EDs) is crucial. This study compares the prognostic value of the National Early Warning Score-2 (NEWS-2), Modified Early Warning Score (MEWS), and Peripheral Perfusion Index (PPI).

**Aims:**

To compare the performance of NEWS-2, MEWS, and PPI in predicting 30-day mortality among critically ill patients admitted to the intensive care unit.

**Methods:**

A prospective, observational study (June–October 2024) included 306 critically ill patients. NEWS-2, MEWS, and PPI were recorded upon presentation. The primary outcome was 30-day mortality. ROC analysis was used to determine the area under the curve (AUC), sensitivity, and specificity.

**Results:**

The 30-day mortality rate was 29.7% (n = 91). Significant differences were found in all scores between survivors and non-survivors (p < .001). NEWS-2 demonstrated the highest discrimination (AUC 0.666; 95% CI 0.598–0.733), followed by MEWS (AUC 0.643) and PPI (AUC 0.633). While NEWS-2 (cutoff ≥ 6) provided the highest sensitivity at 71.4%, the PPI (cutoff ≤ 1.15) exhibited the highest specificity at 78.6%.

**Conclusions:**

NEWS-2 remains the strongest single predictor for 30-day mortality. However, the PPI offers high specificity and reflects microcirculatory impairment, making it a valuable complementary tool alongside established early warning scores rather than a replacement.

## Introduction

Emergency departments (EDs) are some of the most critical components of healthcare systems. Physicians working in EDs are under constant pressure to quickly assess patients and identify cases at risk of deterioration. Because clinical deterioration can often be detected early based on changes in vital signs, the early identification of high-risk patients plays a critical role in reducing mortality and morbidity [1–3]. To that end, early warning scores have been developed and are increasingly used in clinical practice.

One early warning score, the National Early Warning Score (NEWS), was developed by the Royal College of Physicians in 2012 and updated in 2017 with the name “NEWS-2.” NEWS-2 includes parameters such as respiratory rate, pulse, systolic blood pressure, oxygen saturation, oxygen support, level of consciousness, and body temperature. The literature reports that NEWS-2 has high discriminatory power in predicting serious clinical outcomes such as mortality, intensive care unit admission, and the need for mechanical ventilation [4–6].

Another early warning score, the Modified Early Warning Score (MEWS), was defined in 1999 and includes the parameters of respiratory rate, heart rate, systolic blood pressure, body temperature, and level of consciousness. Its ease of calculation and lack of need for laboratory data are important advantages. Even so, some studies have shown that MEWS is not as strong as NEWS-2 in predicting mortality [7–9].

In recent years, studies on early warning scores and the noninvasive assessment of microcirculation have proliferated. Among the latter, the Peripheral Perfusion Index (PPI) is a parameter derived from the pulse oximetry signal that reflects the pulsatile component of peripheral circulation. Low PPI values indicate impaired tissue perfusion and have been associated with mortality in critical conditions such as sepsis and shock. The PPI’s rapid, noninvasive, and continuous measurability makes it a potentially valuable complementary tool in clinical decision-making in EDs [10–12].

Against that background, the aim of our study was to compare the performance of NEWS-2, MEWS, and PPI in predicting 30-day mortality among critically ill patients who present at EDs. The literature reports that NEWS-2 is a stronger predictor than MEWS, while the PPI may provide additional prognostic value. Our study’s results are significant in that they capture the effectiveness of those three methods together in the same patient group.

## Material and methods

Our prospective, single-center observational study included critically ill patients who presented to the ED of a tertiary care hospital between June 2024 and October 2024 and were admitted to the intensive care unit. The study was approved by the local ethics committee (HRÜ/24.08.37) and conducted in accordance with the Declaration of Helsinki. Patients were eligible for inclusion if they were assigned a red triage code at ED presentation, were subsequently admitted to the ICU from the ED, and provided written informed consent themselves or through a relative. Our ED uses the national color-coded triage approach commonly applied in Turkish emergency departments, in which patients are categorized into green, yellow, and red areas according to urgency and clinical severity. In this system, the red triage category refers to patients with unstable or life-threatening conditions requiring immediate evaluation and treatment. Inclusion was not based on triage code alone; ICU admission was determined after clinical evaluation by the emergency and intensive care teams for patients with acute critical illness requiring close monitoring and/or advanced supportive care. These patients typically had evidence of significant clinical deterioration, such as hemodynamic instability, respiratory distress or failure, altered mental status, or other conditions warranting intensive care follow-up. Exclusion criteria included patients presenting with trauma or cardiac arrest, who were < 18 years old, who were pregnant, and who did not provide written consent.

In a power analysis conducted prior to the study, the required sample size for the primary endpoint was calculated to be 98. Ultimately, 306 patients who met the mentioned criteria during the study period were included in the study. No additional interventions beyond routine medical care were performed on the patients during the study, and only data obtained from the hospital’s record system, ED records, and, when necessary, statements from the patient or family were used. All data were collected under the supervision of at least one emergency medicine specialist.

The collected variables included demographic data (i.e., age, sex), oxygen support status, pulse rate (beats per minute), systolic and diastolic arterial pressure (mmHg), body temperature (°C), respiratory rate (breaths per minute), oxygen saturation (SpO_2_, %), level of consciousness, Glasgow Coma Scale (GCS) score, and partial carbon dioxide pressure (pCO_2_) obtained from arterial blood gas (ABG) analysis. ABG samples were taken within the first 5 min after admission. The primary outcome was 30-d mortality following intensive care admission. Data indicating mortality were verified through hospital information systems, the official death notification system, and telephone contact with the patient or their relatives 30 d after admission. Patients who were discharged from the hospital in good health within 30 d or who were still hospitalized on the 30th day were included in the survivor group.

PPI measurements were taken within the first 5 min following the patient’s presentation to the ED, in the resuscitation area at a constant ambient temperature (i.e., 24 °C), in the supine position, and from the second finger of the right hand. Measurements were recorded in real time using a portable pulse oximeter (Masimo Rad-97 Pulse CO-Oximeter, Masimo Corp., Irvine, CA) and a fingertip transcutaneous probe (RD rainbow Neo 8λ SpCO., Irvine, CA). Personnel performing the measurements were trained in using the device and the measurement protocol, and the devices were checked prior to use in accordance with the manufacturer’s recommendations.

NEWS-2 score was calculated based on seven parameters: respiratory rate (breaths per minute), oxygen saturation (SpO_2_,%), oxygen therapy status (i.e., receiving or not receiving), systolic blood pressure (mmHg), pulse (beats per minute), level of consciousness (i.e., alert or CVPU), body temperature (°C). Separate calculations were performed for patients with and without hypercapnic respiratory failure when calculating the saturation score. *Hypercapnia* was defined as arterial pCO_2_ > 45 mmHg. The MEWS score was calculated according to the relevant literature criteria, and scores ≥ 5 were considered to indicate critical illness.

Statistical analyses were performed using SPSS version 22.0 (IBM Corp., Armonk, NY, USA). The distributions of continuous variables were assessed using the Kolmogorov–Smirnov test, and descriptive statistics are presented as *M* ± *SD* or median (i.e., min.–max.). Independent sample *t* tests or Mann–Whitney *U* tests were used for comparisons between two groups, while chi-square tests were used for categorical variables. Receiver operator characteristic (ROC) curves were used to predict the primary outcome, the area under the curve (AUC) was calculated, and the optimal threshold values were determined using the Youden index. In all tests, *p* < 0.05 was accepted as the two-tailed level of significance.

## Results

A total of 306 patients were included in the study. The median age of the study population was 72 years (18–99), and 48.7% were male, while 51.3% were female. The 30-d mortality rate was determined to be 29.7% (*n* = 91). No significant differences emerged in demographic characteristics between patients who developed mortality and ones who survived; however, significant differences were observed in the patients’ scores.

The MEWS score was 2.73 ± 1.64 among survivors and 3.84 ± 2.27 among ones who died. That difference was statistically significant (*p* < 0.001), as was the difference betweentheirNEWS-2 scores(survivors: 5.47 ± 3.67; deaths: 7.79 ± 4.02). PPI scores were 3.07 ± 2.48 among survivors and 2.11 ± 1.97 among ones who died, and the difference was statistically significant (*p* < 0.001). All those summary findings appear in Table 1.

**Table 1:** Demographic and clinical characteristics of the study’s population

Variable	Total (*n* = 306)	Survivors (*n* = 215)	Deaths (*n* = 91)	*p*
Age, years, median (min–max)	72 (18–99)	70 (18–99)	74 (22–99)	**0.015**
Sex (M/F)	149/157	100/115	49/42	0.241
MEWS, median (min–max)	3 (0–10)	3 (0–9)	4 (0–10)	** < 0.001**
NEWS-2, median (min–max)	6 (0–16)	5 (0–15)	8 (0–16)	** < 0.001**
PPI, median (min–max)	2.10 (0.06–13.0)	2.30 (0.17–13.0)	1.50 (0.06–8.90)	** < 0.001**

MEWS = Modified Early Warning Score; NEWS-2 = National Early Warning Score 2; PPI = Peripheral Perfusion Index

Regarding cutoff values, a threshold of ≥ 6 for NEWS-2 provided the highest sensitivity at 71.4% and highest specificity at 53.5%. A threshold of ≥ 4 for MEWS showed 50.5% sensitivity and 74.0% specificity. For the PPI, the ≤ 1.15 cutoff value provided high specificity (78.6%) and 44.0% sensitivity. Those cutoff values and performance metrics are summarized in Table 2.

**Table 2:** Receiver operating characteristic analysis and performance metrics

	AUC	95% CI	*p*	Cutoff	Sensitivity	Specificity	PPV	NPV
NEWS‑2	.666	0.598, 0.733	<.001	≥ 6	71.4%	53.5%	39.4%	81.6%
MEWS	.643	0.572, 0.714	<.001	≥ 4	50.5%	74.0%	45.1%	77.9%
PPI	.633	0.563, 0.703	<.001	≤ 1.15	44.0%	78.6%	46.5%	76.8%

AUC = area under curve; CI = confidence interval; PPV = Positive Predictive Value; NPV = Negative Predictive Value; NEWS-2 = National Early Warning Score 2; MEWS = Modified Early Warning Score; PPI = Peripheral Perfusion Index

**Fig. 1:**
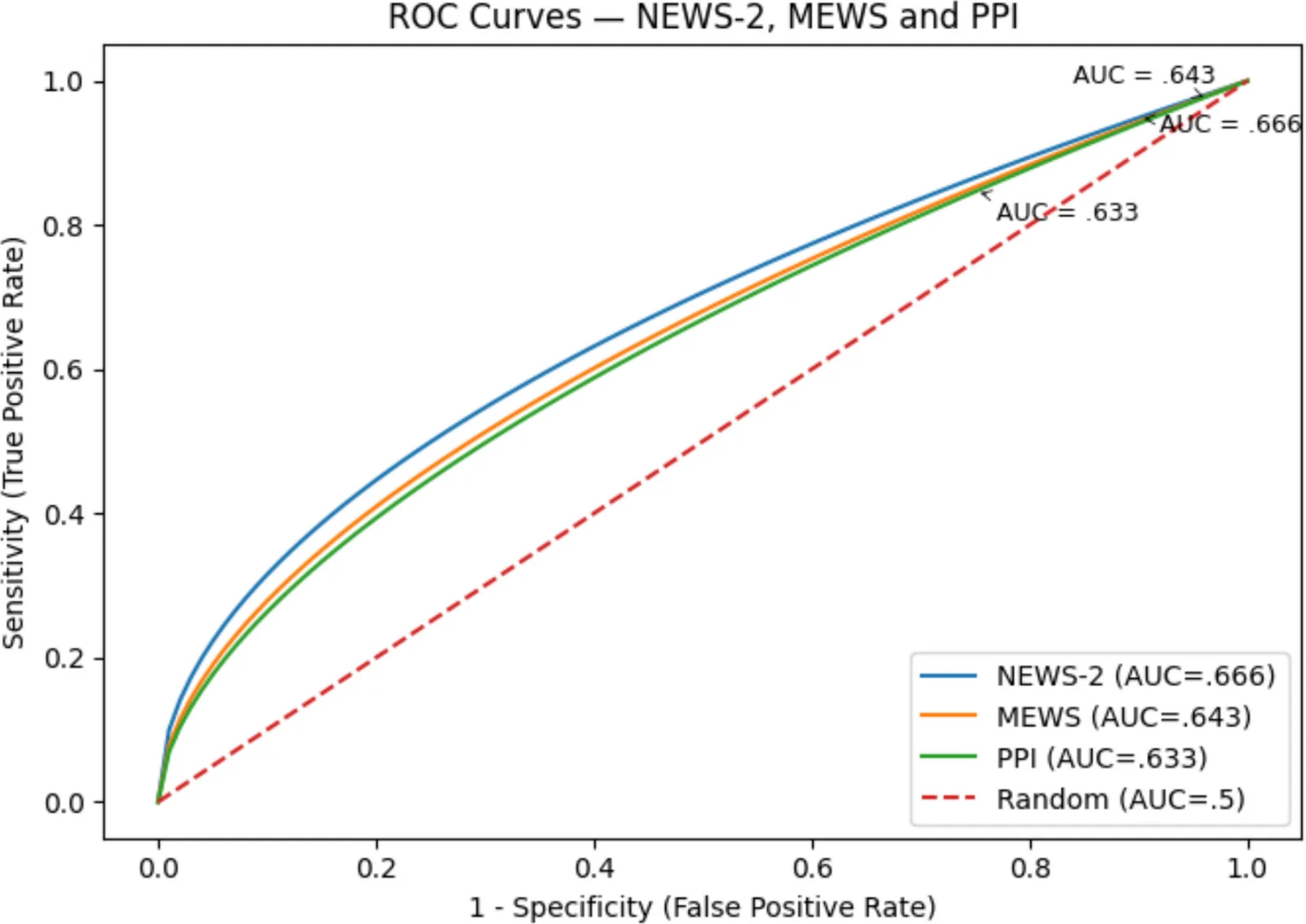
ROC Analysis for the National Early Warning Score 2 (NEWS-2), Modified Early Warning Score (MEWS), and Peripheral Perfusion Index (PPI) *Note*. AUC = area under curve; PPV = Positive Predictive Value; NPV = Negative Predictive Value

According to ROC curve analysis, the AUC of NEWS-2 in predicting 30-d mortality was 0.666 (95% CI: 0.598, 0.733). The AUC of MEWS was 0.643 (95% CI: 0.572, 0.714), and the AUC of PPI was 0.633 (95% CI: 0.563, 0.703), as shown in Fig. 1.

## Discussion

In our study, we compared the performance of MEWS, NEWS-2, and PPI scores in predicting 30-day mortality among 306 critically ill patients presenting to the ED. Although NEWS-2 had a higher AUC than MEWS and PPI, its discriminative performance remained modest overall. Therefore, rather than indicating strong predictive accuracy, our findings suggest that NEWS-2 performed relatively better than the other evaluated parameters within this specific cohort, while still showing limited overall ability to discriminate 30-day mortality risk.

A review of the literature shows that the prognostic value of those scores varies according to different patient groups and follow-up periods. Large meta-analyses have revealed that NEWS-2 has an average AUC value of approximately 0.80 in predicting 30-d mortality and provides balanced sensitivity/specificity ratios [13]. In our study, however, the AUC of NEWS-2 was 0.666. Several factors may explain this difference. First, our study included only red triage patients who were critically ill and subsequently admitted to the ICU from the ED, representing a relatively narrow, high-risk, and partially homogeneous population. In such a cohort, baseline physiologic derangement may already be common, and the overall spectrum of illness severity may be compressed, reducing the discriminative ability of NEWS-2 compared with studies including broader ED or prehospital populations. Second, despite this high-acuity selection, the study population remained clinically heterogeneous, and different underlying disease processes may have influenced the prognostic performance of early warning scores. Third, our primary endpoint was 30-day mortality rather than immediate deterioration or short-term adverse outcomes; therefore, mortality may have been affected not only by the physiologic status at ED presentation but also by subsequent ICU management, treatment response, comorbid conditions, and in-hospital complications. Finally, NEWS-2 was calculated using a single set of admission data, whereas serial measurements may better capture dynamic clinical deterioration and potentially improve prognostic accuracy.

A prospective study conducted in a prehospital setting has shown that the PPI is superior to NEWS score in predicting discharge and 24-hmortality [14]. Another study conducted in Turkey has indicated that PPI scores showed significant differences in discharge and admission decisions in the ED and provided discriminatory power similar to ESI scores for discharge from the ED [15]. It has also been shown that the PPI particularly reflects microcirculatory impairment and may change before classic vital signs [16]. However, a large systematic review of prehospital early warning scores revealed that the AUC values of NEWS-2 and similar scores ranged from 0.68 to 0.82 in predicting 30-d mortality, with both under- and over-triage risks observed [17]. Those findings suggest that the PPI may be a strong prognostic marker in some patient groups, particularly in the early period, for it measures microcirculation parameters that reflect hemodynamic deterioration.

However, each study has had a different patient profile and used different PPI threshold values. In our study, applying a PPI cutoff value of 1.15 yielded low sensitivity (44.0%) but relatively high specificity (78.6%). This pattern suggests that the PPI may be less suitable as a screening tool, but more useful as a “red flag” marker that alerts clinicians to increased mortality risk when measured at low levels. In that sense, a low PPI value may help identify a subgroup of patients requiring closer attention, even if normal or higher values cannot reliably exclude risk. Therefore, rather than replacing NEWS-2, the PPI may be more appropriately interpreted as a complementary warning parameter alongside clinical assessment and other early warning scores. By contrast, the PPI’s dynamic, noninvasive nature may benefit clinicians, particularly in situations in which peripheral vasoconstriction is an early determinant. As an indicator, the PPI measures the ratio of pulsatile and non pulsatile pulse wave components and reflects the perfusion of peripheral tissues [16]. Because microcirculation is sensitive to hemodynamic deterioration, PPI scores may change before classic vital signs. Nevertheless, external factors such as ambient temperature, probe placement, and peripheral vasoconstriction can affect PPI values. Therefore, instead of replacing NEWS-2, the PPI should be considered as a complementary tool alongside clinical assessment and other early warning scores.

### Limitations

Our results have some limitations. For one, our study’s single-center design and relatively small sample size may limit the generalizability of our results. For another, PPI measurements were taken only at the time of presentation, and serial measurements may be more meaningful in capturing dynamic changes. Certain patient groups, including trauma, cardiac arrest, pediatric, and pregnant patients, were excluded from our study in order to obtain a more clinically homogeneous cohort. However, this selection may also have introduced a degree of selection bias by limiting the analysis to a specific subgroup of critically ill ED patients. Trauma and cardiac arrest patients often have distinct physiologic patterns, mortality mechanisms, and resuscitation priorities, and the prognostic performance of NEWS-2, MEWS, and PPI may differ in those populations. Therefore, our findings should be interpreted primarily in critically ill non-trauma, non-cardiac arrest adults and may not be fully generalizable to the entire emergency department population. Furthermore, although PPI measurements were standardized as much as possible, PPI values may still be influenced by factors affecting peripheral circulation, including finger selection, ambient temperature, peripheral vasoconstriction, vasoactive drug use, and underlying peripheral arterial disease. These factors may alter peripheral perfusion independently of systemic hemodynamic status and could therefore have affected the measured PPI values. Since such potential confounders were not evaluated separately, their effect on the prognostic performance of PPI should be considered when interpreting our findings. Last, because only 30-dmortality outcomes were evaluated, different follow-up periods or composite endpoints may alter the prognostic performance of the PPI and NEWS-2.

## Conclusion

In sum, although NEWS-2 showed better discriminatory performance than MEWS and PPI in this cohort, its overall predictive ability for 30-day mortality was modest. PPI may still be a valuable complementary marker, particularly because of its relatively high specificity, and may help identify patients at increased risk when interpreted together with NEWS-2 and clinical assessment. The PPI’s ability to reflect microcirculation and its ease of measurement with existing pulse oximetry devices offer advantages, particularly in detecting hemodynamic deterioration early on. Future multicenter studies with standardized measurement protocols and serial PPI assessments could more clearly demonstrate the parameter’s prognostic value in various patient populations. Instead of relying on a single score or parameter, the use of the PPI in conjunction with NEWS-2 and other clinical indicators may optimize risk stratification.

## Data Availability

The datasets generated and/or analyzed during the current study are available from the corresponding author on reasonable request.
